# On the Shape of Limit Cycles That Bifurcate from Isochronous Center

**DOI:** 10.1155/2014/320406

**Published:** 2014-03-19

**Authors:** Guang Chen, Yuhai Wu

**Affiliations:** Faculty of Science, Jiangsu University, Zhenjiang, Jiangsu 212013, China

## Abstract

New idea and algorithm are proposed to compute asymptotic expression of limit cycles bifurcated from the isochronous center. Compared with known inverse integrating factor method, new algorithm to analytically computing shape of limit cycle proposed in this paper is simple and easy to apply. The applications of new algorithm to some examples are also given.

## 1. Introduction

Many physical, chemical, and biological systems show periodic activity. Mathematically, they can be modeled by limit cycles of vector field. For example, in [1], Van der Pol proved that a closed trajectory of a self-sustained oscillation occurring in a vacuum tube circuit was a limit cycle as defined by Poincaré. The study of limit cycles of real general planar vector field is closely related to Hilbert's 16th Problem. As to the strongly nonlinear oscillation equation *dx*/*dt* = *y*, *dy*/*dt* = *g*(*x*) + *ɛ* *f*(*x*, *y*), in [2], the first two order approximate expressions of limit cycles for small positive parameter *ɛ* were studied by the generalized KBM method, and, in [3], the shape of the limit cycles for moderately large positive parameter *ɛ* was plotted by using the perturbation-incremental method.

In 1881–1886, Poincaré defined a center of planar vector field as an isolated singular point surrounded by a family of periodic orbits. Then one interesting problem is to ask whether limit cycles appear near the periodic orbits in the vicinity of the center as the planar vector field having a center is perturbed, and what are the shapes of these limit cycles if they exist? Literatures [4, 5] have applied the method of inverse integrating factor to analytically compute global shape of the limit cycles bifurcated from analytic isochronous center. The main idea of determining the shape of limit cycles of planar vector field (*P*(*x*, *y*), *Q*(*x*, *y*)) in [4, 5] is to determine function *V*(*x*, *y*) = ∑_*k*=0_^*∞*^ *ε* ^*k*^ *V*_*k*_(*x*, *y*) which satisfies the partial differential equation

(1) $$\begin{array}{c} P\frac{\partial V}{\partial x}+Q\frac{\partial V}{\partial y}=\left(\frac{\partial P}{\partial x}+\frac{\partial Q}{\partial y}\right)V, \end{array}$$

and the limit cycles of planar vector field (*P*(*x*, *y*), *Q*(*x*, *y*)) are implicitly determined by *V*(*x*, *y*) = 0. In other words, if one tries to find analytic expression of limit cycle, one should solve linear partial differential equations recursively. In this paper, a new idea and algorithm are developed to analytically compute the shape of the limit cycles bifurcated from the isochronous center. From Theorem  3.2 in [6], we know that any planar analytic system having isochronous center can be locally transformed into the above linear system $\dot{x}=y$, $\dot{y}=-x$ by analytic variable transformation and time scale. So without losing generality, we consider analytic expression of limit cycle of perturbed planar vector field $\dot{x}=y+ɛU(x,y,ɛ)$, $\dot{y}=-x+ɛV(x,y,ɛ)$. The new algorithm proposed in the paper is based on the following lemmas.

**Lemma 1**

If planar analytic vector field (*P*(*x*, *y*), *Q*(*x*, *y*)) has a limit cycle *r* = *r*(*θ*) surrounding the origin *O*(0,0), then *r*(*θ*) is a periodic function with period 2*π*, where (*x*, *y*) = (*r*cos⁡*θ*, *r*sin*θ*).

**Proof**

From the periodicity of limit cycle and the property of polar coordinate system, we know that the conclusion of the lemma is true.

**Lemma 2 (see [7])**

If *f*(*θ*) is a *C* ^2^ periodic function with period 2*π*, then

(2) $$\begin{array}{c} F\left(\theta \right)=\overset{\theta }{\underset{0}{\int }}f\left(\xi \right)d\xi =g\theta +\varphi \left(\theta \right), \end{array}$$

where *g* = (1/2*π*)∫_0_^2*π*^ *f*(*ξ*)*dξ* and *φ*(*θ*) is a periodic function with period 2*π*.

Further, if *F*(*θ*) is periodic function, then *g* = 0.

**Proof**

For *f*(*ξ*) is a *C* ^2^ periodic function with period 2*π*, so Fourier coefficients of functions *f*(*ξ*) and *f*′(*ξ*) have the following relations:

(3) a0′=1π∫02πf′(ξ)dξ=1π[f(2π)−f(0)]=0,an′=1π∫02πf′(ξ)cos⁡(nξ)dξ=1πf(ξ)cos⁡(nξ)|02π+nπ∫02πf(ξ)sin(nξ)dξ=nbn, n=1,2,…,bn′=1π∫02πf′(ξ)sin(nξ)dξ=1πf(ξ)sin(nξ)|02π−nπ∫02πf(ξ)cos⁡(nξ)dξ=−nan, n=1,2,….

So

(4) |ancos⁡⁡(nθ)+bnsin⁡(nθ)|≤|an|+|bn|=|an′|n+|bn′|n≤12(an′2+bn′2)+1n2.

By applying Bessel inequality, we get

(5) $$\begin{array}{c} \overset{\infty }{\underset{n=1}{\sum }}\left(a_{n}^{\prime 2}+b_{n}^{\prime 2}\right)\leq \frac{1}{\pi }\overset{2\pi }{\underset{0}{\int }}{f^{\prime }}^{2}\left(\xi \right)d\xi . \end{array}$$

By applying comparison test for convergence of series of functions, we get that Fourier series of *f*(*ξ*) is uniformly convergent to *f*(*ξ*) on [0,2*π*]. Rewrite *f*(*θ*) into the following Fourier series:

(6) $$\begin{array}{c} f\left(\theta \right)=\frac{a_{0}}{2}+\overset{\infty }{\underset{n=1}{\sum }}\left(a_{n}\cos\left(n\theta \right)+b_{n}\sin\left(n\theta \right)\right), \end{array}$$

where

(7) $$\begin{array}{c} \frac{a_{0}}{2}=\frac{1}{2\pi }\overset{2\pi }{\underset{0}{\int }}f\left(\xi \right)d\xi =g. \end{array}$$

Integrating both sides of (6) with respect to variable *θ* from 0 to *θ*, we obtain (2) with

(8) $$\begin{array}{c} \varphi \left(\theta \right)=\overset{\infty }{\underset{n=1}{\sum }}\left[\frac{a_{n}}{n}\sin\left(n\theta \right)-\frac{b_{n}}{n}\left(\cos\left(n\theta \right)-1\right)\right]. \end{array}$$

From the integration property of uniformly convergent series, we get that *φ*(*θ*) is a periodic function with period 2*π*.

If *F*(*θ*) is a periodic function with period 2*π*, then we get *g* · (2*π* − 0) + *φ*(2*π*) − *φ*(0) = 0. From *φ*(2*π*) = *φ*(0), we conclude that *g* = 0.

The proof of the lemma is completed.

The main goal of this paper is to develop a new approach for computing analytically the global shape of the bifurcated limit cycles from an isochronous center and the paper is organized as follows. In Section 2, we develop a new algorithm to compute analytic expansion, up to arbitrary order of the parameter *ɛ*, of the limit cycles bifurcated from linear isochronous center. As applications, in Section 3, we compute the analytic expression of the unique limit cycle of the Van der Pol system *x*′ = *y*, *y*′ = −*x* + *ɛ*(1 − *x* ^2^)*y* up to order *o*(*ε* ^7^). In Section 4, we study the analytic expression of the limit cycle bifurcated from a nonlinear isochronous center.

## 2. Asymptotic Expressions of Limit Cycles Bifurcated from the Center of *x*′ = *y*, *y*′ = −*x*

Consider the following planar system:

(9) x′ =y+∑k=1∞εkpk(x,y)≡y+ɛU(x,y,ɛ),y′ =−x+∑k=1∞εkqk(x,y)≡−x+ɛV(x,y,ɛ),

where *p*_*k*_(*x*, *y*) and *q*_*k*_(*x*, *y*) are both analytic functions, *p*_*k*_(0,0) = *q*_*k*_(0,0) = 0, *k* = 1,2,…, and *ɛ* is a small real parameter. System (9) has an isochronous center at the origin when *ɛ* = 0. As usual, the prime denotes derivative with respect to variable *t*. System (9) for *ɛ* = 0 is called the unperturbed system, while system (9) for *ɛ* ≠ 0 is called the perturbed one. Then the problem of studying shape of limit cycles bifurcated from isochronous center is to determine the number and analytic expansions of the families of limit cycles which emerge from the periodic orbits of the unperturbed system as the parameter *ɛ* is varied.

The main idea of computing asymptotic expression of limit cycles of system (9) is the following.

Firstly, we make a polar coordinates transformation *x* = *r*cos⁡*θ*, *y* = *r*sin*θ* to system (9). By eliminating the variable *t*, we obtain

(10) $$\begin{array}{c} \frac{dr}{d\theta }=\frac{ɛ\left(U\cos\theta +V\sin\theta \right)}{-1+ɛ\left(V\cos\theta -U\sin\theta \right)/r}. \end{array}$$

By noticing that *p*_*k*_(*x*, *y*), *q*_*k*_(*x*, *y*) are both analytic functions, we rewrite system (10) into the following form:

(11) $$\begin{array}{c} \frac{dr}{d\theta }=R_{1}\left(r,\theta \right)ɛ+R_{2}\left(r,\theta \right)\varepsilon^{2}+R_{3}\left(r,\theta \right)\varepsilon^{3}+\cdots , \end{array}$$

where *R*_1_(*r*, *θ*), *R*_2_(*r*, *θ*),… are analytic functions about *r*, cos⁡(*θ*), sin(*θ*).

Secondly, to obtain the polar coordinate form of limit cycles of perturbed system (9), we look for the following analytic solution to (11) as *ɛ* ≠ 0,

(12) $$\begin{array}{c} r\left(\theta \right)=\overset{\infty }{\underset{k=0}{\sum }}\varepsilon^{k}r_{k}\left(\theta \right). \end{array}$$

From Lemma 1, we know that if *r*(*θ*) is limit cycle of system (10), then from the periodicity of *r*(*θ*), we get that *r*_*k*_(*θ*) is periodic function with period 2*π*, where *k* = 0,1, 2,….

Thirdly, substituting (12) into (11) and considering the *k*th order terms of *ɛ* in the obtained system, then we can obtain a series of equations:

(13) $$\begin{array}{c} \frac{dr_{k}}{d\theta }={\tilde{f}}_{k}\left(\theta ,r_{0},r_{1},r_{2},\ldots \right),\;\;k=0,1,2,\ldots . \end{array}$$

As to the formula of ${\tilde{f}}_{k}(\theta ,r_{0},r_{1},r_{2},\ldots )$, we have the following lemma.

**Lemma 3**

Functions ${\tilde{f}}_{k}(\theta ,r_{0},r_{1},r_{2},\ldots )$ obtained in (13) have the following properties:

(14) f~0(θ,r0,r1,r2,…)=0,f~1(θ,r0,r1,r2,…)=f1(r0,θ),f~2(θ,r0,r1,r2,…)=f2(r1,r0,θ),           ⋮f~k(θ,r0,r1,r2,…)=fk(rk−1,rk−2,…,r1,r0,θ),           ⋮

where *f*_*k*_(*r*_*k*−1_, *r*_*k*−2_,…, *r*_1_, *r*_0_, *θ*) is analytic function about *r*_*k*−1_, *r*_*k*−2_,…, *r*_1_, *r*_0_, cos⁡(*θ*), sin(*θ*), *k* = 0,1, 2,….

**Proof**

According to (11) and (12), we get that

(15) $$\begin{array}{c} \overset{\infty }{\underset{k=0}{\sum }}\varepsilon^{k}\frac{dr_{k}\left(\theta \right)}{d\theta }=R_{1}\left(r,\theta \right)ɛ+R_{2}\left(r,\theta \right)\varepsilon^{2}+R_{3}\left(r,\theta \right)\varepsilon^{3}+\cdots . \end{array}$$

For *k* = 0, it is easy to get that ${\tilde{f}}_{0}(\theta ,r_{0},r_{1},r_{2},\ldots )=0$.

For *k* = 1, the term on the right hand side of (15) contributing to *ε* ^1^ is *ɛR*_1_(*r*, *θ*). In detail, the constant in the term *R*_1_(*r*, *θ*) determines ${\tilde{f}}_{1}(\theta ,r_{0},r_{1},r_{2},\ldots )$. Noticing that *r*(*θ*) = *r*_0_ + *ɛr*_1_(*θ*) + *ε* ^2^ *r*_2_(*θ*)+⋯, we get that ${\tilde{f}}_{1}(\theta ,r_{0},r_{1},r_{2},\ldots )$ only contains the term *r*_0_. In other words, if function ${\tilde{f}}_{1}(\theta ,r_{0},r_{1},r_{2},\ldots )$ contains variable *r*_*n*_, *n* ≥ 1, then *r*_*n*_ appearing in the terms in the right hand side of (15) at least have term *ε* ^*n*+1^. This is contradiction, for ${\tilde{f}}_{1}(\theta ,r_{0},r_{1},r_{2},\ldots )$ corresponds to *ε* ^1^ term in the right hand side of (15). By using similar analysis, it can be shown that ${\tilde{f}}_{n}(\theta ,r_{0},r_{1},r_{2},\ldots )$ cannot contain term *r*_*i*_, *i* ≥ *n*. Therefore, ${\tilde{f}}_{k}(\theta ,r_{0},r_{1},r_{2},\ldots )=f_{k}(r_{k-1},r_{k-2},\ldots ,r_{1},r_{0},\theta )$, *k* = 0,1, 2,….

The proof of the lemma is completed.

To obtain analytic expression of limit cycle *r* = *r*(*θ*) of system (9), we need to determine *r*_*k*_(*θ*) in (12), *k* = 0,1, 2,…. From Lemma 3, we know that the determinations of *r*_*k*_(*θ*) in (12) are recursive.

### 2.1. Determination of *r*_0_ and the Poincaré-Melnikov Integral

From *dr*_0_/*dθ* = 0, we get *r*_0_(*θ*) ≡ *r*_0_(constant). To determine the constant *r*_0_ in (12), the new approach we adopted is to utilize the expression of *r*_1_(*θ*).

From *dr*_1_/*dθ* = *f*_1_(*r*_0_, *θ*), we obtain

(16) $$\begin{array}{c} r_{1}\left(\theta \right)=\overset{\theta }{\underset{0}{\int }}f_{1}\left(r_{0},\theta \right)d\theta +c_{1}. \end{array}$$

By noting that *f*_1_(*r*_0_, *θ*) is a periodic function, according to Lemma 2, we know

(17) $$\begin{array}{c} \overset{\theta }{\underset{0}{\int }}f_{1}\left(r_{0},\theta \right)d\theta =g_{1}\theta +\varphi_{1}\left(r_{0},\theta \right), \end{array}$$

where $g_{1}=\left(1/2\pi \right)\int_{0}^{2\pi }f_{1}(r_{0},\theta )d\theta \equiv {\bar{g}}_{1}(r_{0})$, and *φ*_1_(*r*_0_, *θ*) is periodic function with period 2*π*.

From Lemma 1, we know that if *r* = *r*(*θ*) is a limit cycle of system (9), then *r*(*θ*) and *r*_1_(*θ*) in (16) are periodic functions, too.

So from Lemma 2, we know $g_{1}={\bar{g}}_{1}(r_{0})=0$. By solving that algebraic equation, we can determine the value of constant *r*_0_.

**Remark 4**

In fact, the function ${\bar{g}}_{1}(r_{0})$ is closely related to the first order Poincare-Melnikov integral of the perturbed system (9) near close orbit of unperturbed system (9)|_*ɛ*=0_.

In detail, ${\bar{g}}_{1}(r_{0})=-\left(1/2\pi r_{0}\right)\oint_{L}q_{1}(x,y)dx-p_{1}(x,y)dy$, where close orbit *L* : *x* ^2^ + *y* ^2^ = *r*_0_^2^. From [8, 9], we know $-2\pi r_{0}{\bar{g}}_{1}(r_{0})$ is the first order Poincare-Melnikov integral. So the zeros of ${\bar{g}}_{1}(r_{0})$ are closely related to the number and position of limit cycles of the perturbed system (9).

It should be pointed out that the function ${\bar{g}}_{1}(r_{0})$ is also closely related to the first order averaging of 1-dimensional 2*π*-periodic differential equation. First order (resp., second order) averaging method to study the existence and number of periodic orbits of planar differential equation is proposed in [10, 11]. The approach of high order averaging method is based on Brouwer degree theory (see [11] for more details).

### 2.2. Determination of *r*_1_(*θ*)

Substitute the value of *r*_0_ into (16); we can obtain expression of *φ*_1_(*r*_0_, *θ*). Thus we obtain

(18) $$\begin{array}{c} r_{1}\left(\theta \right)=\varphi_{1}\left(r_{0},\theta \right)+c_{1}. \end{array}$$

To determine the value of *c*_1_, new algorithm proposed in this paper needs the expression of *r*_2_(*θ*). From *dr*_2_/*dθ* = *f*_2_(*r*_1_, *r*_0_, *θ*), we get

(19) $$\begin{array}{c} r_{2}\left(\theta \right)=\overset{\theta }{\underset{0}{\int }}f_{2}\left(r_{1},r_{0},\theta \right)d\theta +c_{2}. \end{array}$$

From Lemma 2, we know

(20) $$\begin{array}{c} \overset{\theta }{\underset{0}{\int }}f_{2}\left(r_{1},r_{0},\theta \right)d\theta =g_{2}\theta +\varphi_{2}\left(r_{1},r_{0},\theta \right), \end{array}$$

where $g_{2}=\left(1/2\pi \right)\int_{0}^{2\pi }f_{2}(r_{1},r_{0},\theta )d\theta \equiv {\bar{g}}_{2}(c_{1})$.

From the fact that *r*_2_(*θ*) is a periodic function and Lemma 2, we get ${\bar{g}}_{2}(c_{1})=0$.

By solving the above algebraic equation, we determine the value of *c*_1_. Thus we have obtained *r*_1_(*θ*) by (18).

### 2.3. Determination of *r*_*k*_(*θ*)

Assuming that we have obtained the explicit expressions of *r*_0_, *r*_1_(*θ*),…, *r*_*k*−1_(*θ*), now we start to determine *r*_*k*_(*θ*).

From *dr*_*k*_/*dθ* = *f*_*k*_(*r*_*k*−1_, *r*_*k*−2_,…, *r*_1_, *r*_0_, *θ*), we get

(21) $$\begin{array}{c} r_{k}\left(\theta \right)=\overset{\theta }{\underset{0}{\int }}f_{k}\left(r_{k-1},r_{k-2},\ldots ,r_{1},r_{0},\theta \right)d\theta +c_{k}. \end{array}$$

To determine the expression of *r*_*k*_(*θ*) is to determine the value of *c*_*k*_. According to the algorithm proposed in this paper, we resort to the expression of *r*_*k*+1_(*θ*). From *dr*_*k*+1_/*dθ* = *f*_*k*+1_(*r*_*k*_, *r*_*k*−1_,…, *r*_1_, *r*_0_, *θ*), we get

(22) $$\begin{array}{c} r_{k+1}\left(\theta \right)=\overset{\theta }{\underset{0}{\int }}f_{k+1}\left(r_{k},r_{k-1},\ldots ,r_{1},r_{0},\theta \right)d\theta +c_{k+1}. \end{array}$$

From Lemma 2, we know

(23) ∫0θfk+1(rk,rk−1,…,r1,r0,θ)dθ  =gk+1θ+φk+1(rk,rk−1,…,r0,θ),

where

(24) $$\begin{array}{c} g_{k+1}=\frac{1}{2\pi }\overset{2\pi }{\underset{0}{\int }}f_{k+1}\left(r_{k},r_{k-1},\ldots ,r_{0},\theta \right)d\theta \equiv {\bar{g}}_{k+1}\left(c_{k}\right). \end{array}$$

Because *r*_*k*+1_(*θ*) is a periodic function, from Lemma 2, we get that $g_{k+1}={\bar{g}}_{k+1}(c_{k})=0$.

By solving the algebraic equation, we obtain the value of *c*_*k*_; thus we determine *r*_*k*_(*θ*) by (20).

Thus we can compute the shape of limit cycles of system (9) to any given order of *ɛ* explicitly and recursively.

## 3. The Shape of Limit Cycle of Van der Pol System

In this section we will apply the method just described in the above section to compute the analytic expansion of the unique limit cycle of the Van der Pol system

(25) $$\begin{array}{c} x^{\prime }=y,\;\;y^{\prime }=-x+ɛ\left(1-x^{2}\right)y \end{array}$$

up to *o*(*ε* ^7^).

First we make a polar coordinates transformation *x* = *r*cos⁡(*θ*)*y* = *r*sin(*θ*) to system (25) and eliminate *t*; then we can obtain

(26) $$\begin{array}{c} \frac{dr}{d\theta }=\frac{ɛr\left(1-r^{2}\cos^{2}\theta \right)\sin^{2}\theta }{-1+ɛ\left(1-r^{2}\cos^{2}\theta \right)\sin\theta \;\cos\theta }. \end{array}$$

Assume *r*(*θ*) = ∑_*k*=0_^*∞*^ *ε* ^*k*^ *r*_*k*_(*θ*) is the polar coordinates form of the limit cycles of (25) and substitute it into (26). By comparing first eight coefficients of terms *ε* ^*k*^, *k* = 1,2,…, 8 in both sides of the above equation, we get

(27) dr0dθ=0,dr1dθ=−r0(1−r02cos⁡2θ)sin2θ≡f1(r0,θ),dr2dθ=((−1+r02  cos⁡2θ)r1+2r02r1cos⁡2θ)sin2θ+(−1+r02cos⁡2θ)r0  ×sin2θ(−cos⁡θsinθ+r02sinθ cos⁡3θ)≡f2(r1,r0,θ),⋮dr8dθ≡f8(r7,r6,…,r0,θ).

Here for long expressions, the formula of *f*_*k*_(*r*_*k*−1_,…, *r*_1_, *r*_0_, *θ*),  *k* = 3,4,…, 8 is omitted.

From *dr*_0_/*dt* = 0, we get that *r*_0_ is arbitrary constant.

To determine the value of *r*_0_, we compute the following expression of *r*_1_(*θ*):

(28) $$\begin{array}{c} r_{1}\left(\theta \right)=\overset{\theta }{\underset{0}{\int }}f_{1}\left(r_{0},\theta \right)d\theta +c_{1}=g_{1}\theta +\varphi_{1}\left(r_{0},\theta \right)+c_{1}, \end{array}$$

where

(29) g1=12π∫02πf1(r0,θ)dθ=−r0(1−r02cos⁡2θ)sin2θ dθ=18r03−12r0.

Because *g*_1_ = 0, so we get *r*_0_ = 2.

Substitute *r*_0_ = 2 into (28); we obtain

(30) r1(θ)=∫0θf1(2,θ)dθ+c1=2cos⁡θ sinθ−2 cos⁡3θ sinθ+c1.

To determine the value of *c*_1_, we compute the expression of *r*_2_(*θ*):

(31) $$\begin{array}{c} r_{2}\left(\theta \right)=\overset{\theta }{\underset{0}{\int }}f_{2}\left(r_{1},r_{0},\theta \right)d\theta +c_{2}=g_{2}\theta +\varphi_{2}\left(r_{1},r_{0}\right)+c_{2}, \end{array}$$

where

(32) g2=12π∫02πf2(2cos⁡θ sinθ−2 cos⁡3θsinθ+c1,2,θ)dθ=c1.

Because *g*_2_ = 0, so we get *c*_1_ = 0.

Thus explicit expression of *r*_1_(*θ*) is given by (30) with *c*_1_ = 0.

Substitute *r*_1_(*θ*) and *r*_0_ = 2 into (31); we obtain

(33) r2(θ)=∫0θf2(r1,r0,θ)dθ+c2=2 cos⁡2θ−232 cos⁡4θ+493 cos⁡6θ−7 cos⁡8θ+16+c2.

In a similar way, we determine the value of *c*_2_ and obtain following results:

(34) r2(θ)=2cos⁡2θ−232cos⁡4θ+493cos⁡6θ−7cos⁡8θ+1796,r3(θ)=12304(486cos⁡θ+544cos⁡(3θ)−24cos⁡(5θ)−621cos⁡(7θ)−297cos⁡(9θ))sin3θ,r4(θ)=−44336cos⁡14θ+7154cos⁡16θ−1577552960−850211728cos⁡6θ+2150518cos⁡12θ+1796cos⁡2θ+203747576cos⁡8θ−2815330cos⁡10θ−10451152cos⁡4θ,r5(θ)=−41994096cos⁡9(2θ)sin(2θ)+110512288cos⁡8(2θ)× sin(2θ)−1623746080cos⁡6(2θ)sin(2θ)+412037921600sin(2θ)cos⁡4(2θ) −105841518400cos⁡2(2θ)sin(2θ)+62397733177600 sin(2θ)+16948cos⁡7(2θ) ×sin(2θ)−1820349152cos⁡(2θ)sin(2θ)+3047913824sin(2θ)cos⁡3(2θ)−797999184320cos⁡5(2θ)sin(2θ),r6(θ)=−4467994977414400cos⁡7(2θ)−19738393398131200cos⁡3(2θ)+1635728355296000cos⁡5(2θ)−226116384cos⁡11(2θ)−10462939176947200+207757442368cos⁡9(2θ)+21585265116588800×cos⁡6(2θ)−5200332768cos⁡12(2θ)+539326542080×cos⁡(2θ)+166171249132710400cos⁡2(2θ)−8240753589824cos⁡8(2θ)+12241716384cos⁡10(2θ)−1624125463265420800cos⁡4(2θ),r7(θ)=−8413562664072926264320000cos⁡2(2θ)sin(2θ)+29801239711796480cos⁡9(2θ)sin(2θ)+3520666153743178240  cos⁡8(2θ)sin(2θ)−141663013373612672000cos⁡6(2θ)sin(2θ)+21042801308811300561920000sin(2θ)cos⁡4(2θ)+334305131072cos⁡13(2θ) sin(2θ)+260015393216×cos⁡12(2θ) sin(2θ)−7451287589824cos⁡11(2θ)sin(2θ) −62380992211840cos⁡10(2θ)sin⁡(2θ)+28369294256323410114560000sin⁡(2θ)−53313579120643840×cos⁡7(2θ)sin(2θ)+876309326214400cos⁡(2θ) sin(2θ)−58357580831592524800sin(2θ)cos⁡3(2θ)+741891643575308416000×cos⁡5(2θ)sin(2θ).

So the asymptotic expansion of limit cycle of system (25) for *ɛ* > 0 and small is the following:

(35) $$\begin{array}{c} r\left(\theta \right)=\overset{7}{\underset{k=0}{\sum }}\varepsilon^{k}r_{k}\left(\theta \right)+o\left(\varepsilon^{7}\right). \end{array}$$

**Figure 1:**
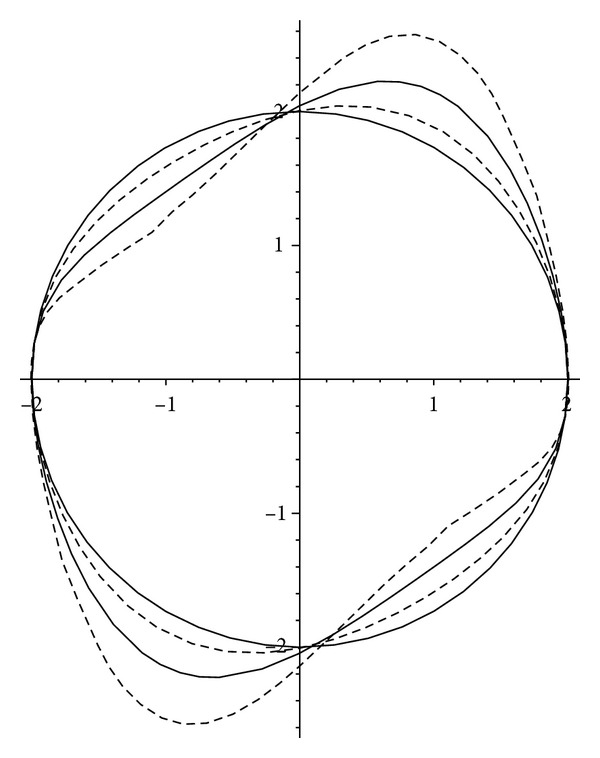
The periodic orbit *x* ^2^ + *y* ^2^ = 4 and the analytical approximations of the limit cycle of Van der Pol system (25), for the values *ɛ* = 0, (1/5), (1/2), (9/10).

The first seven terms in the above expansion of *r*(*θ*) are similar to the ones given in Section 3 of [4]. Here we present the expression of *r*_7_(*θ*) obtained in our method which was omitted in [4] for its long expression. By applying expansion (35), the shapes of limit cycles of Van der Pol system (25) for the values of *ɛ* = 0, (1/5), (1/2), (9/10) are plotted in Figure 1. The periodic orbit *x* ^2^ + *y* ^2^ = 4 of system (25) for *ɛ* = 0 is drawn in solid line, the limit cycle of system (25) for *ɛ* = 1/5 is drawn in dashed line, the limit cycle of system (25) for *ɛ* = 1/2 is drawn in solid line, and the limit cycle of system (25) for *ɛ* = 9/10 is drawn in dotted line.

## 4. The Shape of Limit Cycle of Perturbations of a System Having Nonlinear Isochronous Center 

Consider the following perturbed system:

(36) x′=−y+2xy−2y3+ɛ(x3−3x),y′=x−y2.

From [6], we know that as *ɛ* = 0, nonlinear system (36) has isochronous center *O*(0,0). To utilize new algorithm introduced in Section 2 to study the number and shape of limit cycles of perturbed system (36), we first apply the following analytic variable transformation:

(37) $$\begin{array}{c} x=u+v^{2},\;\;y=v,\;\;t=-\tau \end{array}$$

to system (36) and get

(38) dudτ=v+ɛ(3u+3v2−(u+v2)3),dvdτ=−u.

### 4.1. The Shape of Limit Cycle of the Perturbed System (38)

In this subsection we start to compute the analytic expansion of the limit cycle of the perturbed system (38) to the second order of *ɛ*.

First let *u* = *r*cos⁡*θ*, *v* = *r*sin*θ*; then system (38) is transformed into the following polar coordinate form:

(39) drdθ=ɛ(3rcos⁡θ+3r2sin2θ−(rcos⁡θ+r2sin2θ)3)cos⁡θ×(−1+ɛ(−3cos⁡θ−3r sin2θ+(cos⁡θ+r sin2θ)×(rcos⁡θ+r2sin2θ)2)sinθ)−1.

According to similar process in Section 3, we get

(40) $$\begin{array}{c} g_{1}=\frac{3}{16}r_{0}\left(r_{0}^{4}+2r_{0}^{2}-8\right). \end{array}$$

By solving *g*_1_ = 0, we get the positive solution $r_{0}=\sqrt{2}$.

From $\left(d(-2\pi r_{0}g_{1})/dr_{0}\right){|}_{r_{0}=\sqrt{2}}=-9\sqrt{2}\pi <0$ and [9], we conclude that as *ɛ* > 0, system (38) has a stable limit cycle, denoted by Γ_1_, near the close curve *u* ^2^ + *v* ^2^ = 2 on the phase plane.

By applying the algorithm described in Section 2 to system (39), we get the asymptotic expansion of stable limit cycle Γ_1_ of system (38),

(41) $$\begin{array}{c} r\left(\theta \right)=r_{0}+ɛr_{1}\left(\theta \right)+\varepsilon^{2}r_{2}\left(\theta \right)+O\left(\varepsilon^{3}\right), \end{array}$$

where *r*_0_, *r*_1_(*θ*), *r*_2_(*θ*) are given in the following:

(42) r0=2,r1(θ)=135sin(26+702 cos⁡5θ−1052 cos⁡3θ−22 cos⁡2θ−40 cos⁡6θ+36 cos⁡4θ),r2(θ)=−91435cos⁡3θ+14858175cos⁡5θ+17408105cos⁡9θ−718849cos⁡7θ+1927cos⁡13θ−116811cos⁡11θ−208492 cos⁡14θ−258352 cos⁡12θ+123121752×cos⁡10θ−8367702 cos⁡8θ+1037142 cos⁡6θ−9271402 cos⁡4θ−269352 cos⁡2θ+11303519408002.

**Figure 2:**
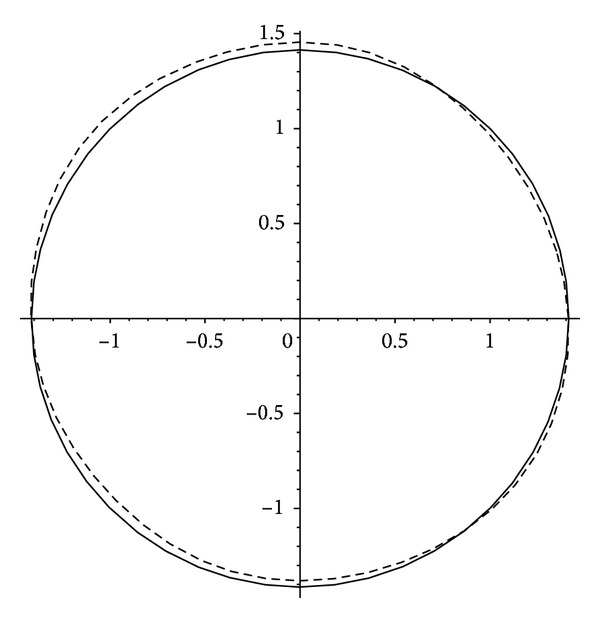
The periodic orbit *u* ^2^ + *v* ^2^ = 2 for *ɛ* = 0 and the analytical and numerical approximations of the limit cycle for *ɛ* = 1/20 of system (38).

In Figure 2 we illustrate the shape of the limit cycle Γ_1_ of the system (38) by using formula (41) for the values *ɛ* = 1/20. The periodic orbit *u* ^2^ + *v* ^2^ = 2 of system (38) for *ɛ* = 0 is drawn in solid line, and the limit cycle Γ_1_ is drawn in dash line. We have also plotted the limit cycle Γ_1_ for the value *ɛ* = 1/20 by using the Runge-Kutta method in Figure 2. The close curve obtained numerically coincides with the one obtained analytically and we cannot distinguish between them with the eyes.

### 4.2. The Shape of Limit Cycle of the Original Perturbed System (36)

In this subsection, we give the analytic expansion of the limit cycle of perturbed system (36) to the second order of *ɛ*.

Rewrite the limit cycle Γ_1_ of system (38) into the following parametric form:

(43) $$\begin{array}{c} u=r\left(\theta \right)\cos\theta ,\;\;v=r\left(\theta \right)\sin\theta , \end{array}$$

where *r*(*θ*) is given in (41).

Thus from analytic transformation and time scale (37), corresponding to Γ_1_, we obtain that limit cycle of the system (36) for *ɛ* = (1/20) is unstable and its parametric form is the following:

(44) x=u+v2=r(θ)cos⁡θ+(r(θ)sinθ)2,y=v=r(θ)sinθ.

**Figure 3:**
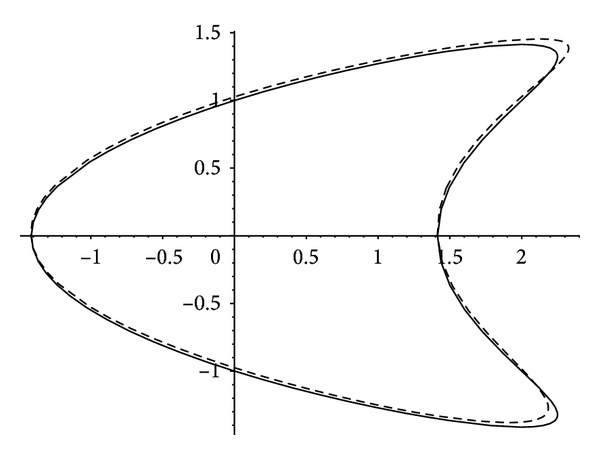
The periodic orbit for *ɛ* = 0 and the analytical and numerical approximations of the limit cycle for *ɛ* = 1/20 of system (36).

The shape of limit cycle of the system (25) for *ɛ* = 1/20 is plotted by using formula (44) in Figure 3.

In Figure 3, the periodic orbit (*x* − *y* ^2^)^2^ + *y* ^2^ = 2 of unperturbed system (36) for *ɛ* = 0 is drawn in sold line, and the limit cycle of the perturbed system (36) for *ɛ* = 1/20 is drawn in dash line.
